# Assessing refugee healthcare needs in Europe and implementing educational interventions in primary care: a focus on methods

**DOI:** 10.1186/s12914-018-0150-x

**Published:** 2018-02-08

**Authors:** Christos Lionis, Elena Petelos, Enkeleint-Aggelos Mechili, Dimitra Sifaki-Pistolla, Vasiliki-Eirini Chatzea, Agapi Angelaki, Imre Rurik, Danica Rotar Pavlic, Christopher Dowrick, Michel Dückers, Dean Ajdukovic, Helena Bakic, Elena Jirovsky, Elisabeth Sophie Mayrhuber, Maria van den Muijsenbergh, Kathryn Hoffmann

**Affiliations:** 1 Clinic of Social and Family Medicine, School of Medicine, University of Crete, University Campus, Voutes, Heraklion, 70013 Crete, GR Greece; 2 Department of Family and Occupational Medicine, Faculty of Public Health, University of Debrecen, Debrecen, Hungary; 3 Department of Family Medicine, Medical Faculty, University of Ljubljana, Ljubljana, Slovenia; 4 Institute of Psychology Health and Society, University of Liverpool, Liverpool, UK; 5 Netherlands Institute for Health Services Research (NIVEL), Utrecht, the Netherlands; 6 Department of Psychology, Faculty of Humanities and Social Sciences, University of Zagreb, Zagreb, Croatia; 7 Department of General Practice and Family Medicine, Center for Public Health, Medical University of Vienna, Vienna, Austria; 8 Department of Primary Care, University Medical Centre Nijmegen St Radboud, Nijmegen, the Netherlands

**Keywords:** Refugees, Migrants, Migration, Person-centred care, Patient-centred, Integrated care, Interdisciplinary care, Primary care, Capacity

## Abstract

The current political crisis, conflicts and riots in many Middle Eastern and African countries have led to massive migration waves towards Europe. European countries, receiving these migratory waves as first port of entry (POE) over the past few years, were confronted with several challenges as a result of the sheer volume of newly arriving refugees. This humanitarian refugee crisis represents the biggest displacement crisis of a generation. Although the refugee crisis created significant challenges for all national healthcare systems across Europe, limited attention has been given to the role of primary health care (PHC) to facilitate an integrated delivery of care by enhancing care provision to refugees upon arrival, on transit or even for longer periods. Evidence-based interventions, encompassing elements of patient-centredness, shared decision-making and compassionate care, could contribute to the assessment of refugee healthcare needs and to the development and the implementation of training programmes for rapid capacity-building for the needs of these vulnerable groups and in the context of integrated PHC care. This article reports on methods used for enhancing PHC for refugees through rapid capacity-building actions in the context of a structured European project under the auspices of the European Commission and funded under the 3rd Health Programme by the Consumers, Health, Agriculture and Food Executive Agency (CHAFEA). The methods include the assessment of the health needs of all the people reaching Europe during the study period, and the identification, development, and testing of educational tools. The developed tools were evaluated following implementation in selected European primary care settings.

## Correspondence

### Background

The current political crisis, conflicts and riots in many Middle Eastern and Sub-Saharan countries have led to an increase of massive migration waves towards Europe. In 2015, 1,255,600 first-time asylum seekers applied for international protection in the Member-States (M-Ss) of the European Union (EU), a number more than double compared to the previous year [1]. Most of these people crossed the borders via the Greek and Italian shores in order to reach Western and Northern European countries in search of safety and a better life [2].

The EUR-HUMAN Project focuses on strengthening PHC given this represents the first contact with the POE countries healthcare system for refugees and migrants, and also other countries accepting them at later stages, with the aim to provide affordable, comprehensive, person-centred, culturally appropriate and integrated care for all ages and ailments. However, the focus of the project, was exclusively on refugees, given Europe experiences the peak in the massive waves of refugee influx during the implementation period of the project, although, migrants were also representing target populations for the reported actions. Although reports on refugee needs were available prior to the period of the crisis, no systematic review and analysis of the refugee needs, wishes and preferences at their arrival had been attempted. Many efforts have been undertaken to address refugee health needs, both physical and mental including extensive efforts of non-governmental organisations (NGOs) in Greece and Italy. Nevertheless, such efforts have been disjointed and fragmented, as well as isolated from existing PHC structures and paths of care; an essential parameter for in the facilitating the smooth entry of refugees into the healthcare system and a foreign, on multiple levels, new society. A systematic approach based on evidence for the role of PHC and for the rapid capacity building of this pillar of care has not been previously discussed and studied. Compassionate care is also currently receiving a lot of attention in the context of PHC [3], with compassion being defined as “*a deep awareness of the suffering of another, coupled with the wish to relieve it*” [4].

Although the notions of integration, patient-centredness, comprehensiveness and compassion have received high attention, with reports in the body of scientific literature emerging from all European countries, they still largely remain rhetoric, with a urgent need of evidence-based interventions encompassing these elements. Lack of integrated PHC in many European settings, including Greece, poses additional obstacles when it comes to implementing coordinated action and capacity-building [5].

Most importantly, efforts assessing prior capacity-building actions and systematic efforts leading to evidence-based interventions are scarce in Europe. The European Commission has repeatedly highlighted the need for implemented evidence-based interventions with sound evaluation to inform and facilitate decision-making in terms of healthcare policymaking. This correspondence article reports on methods used within the EUR-HUMAN project for enhancing PHC for refugees through rapid capacity-building actions, including i) the assessment the health needs of all people that have reached Europe in the period of the project implementation independently their legal status with a more focus on refugees in Europe using a refugee-centred approach, ii) the identification, development and testing of tools and educational interventions to give appropriate healthcare to this population group within the frame of European PHC, and iii) the evaluation of tools developed and implemented in selected European primary care settings. The evaluation was aimed at gaining information on how to better facilitate uptake of successful practices across other M-Ss and beyond the duration and scope of the project.

## Methods- the design of the project

**Fig. 1:**
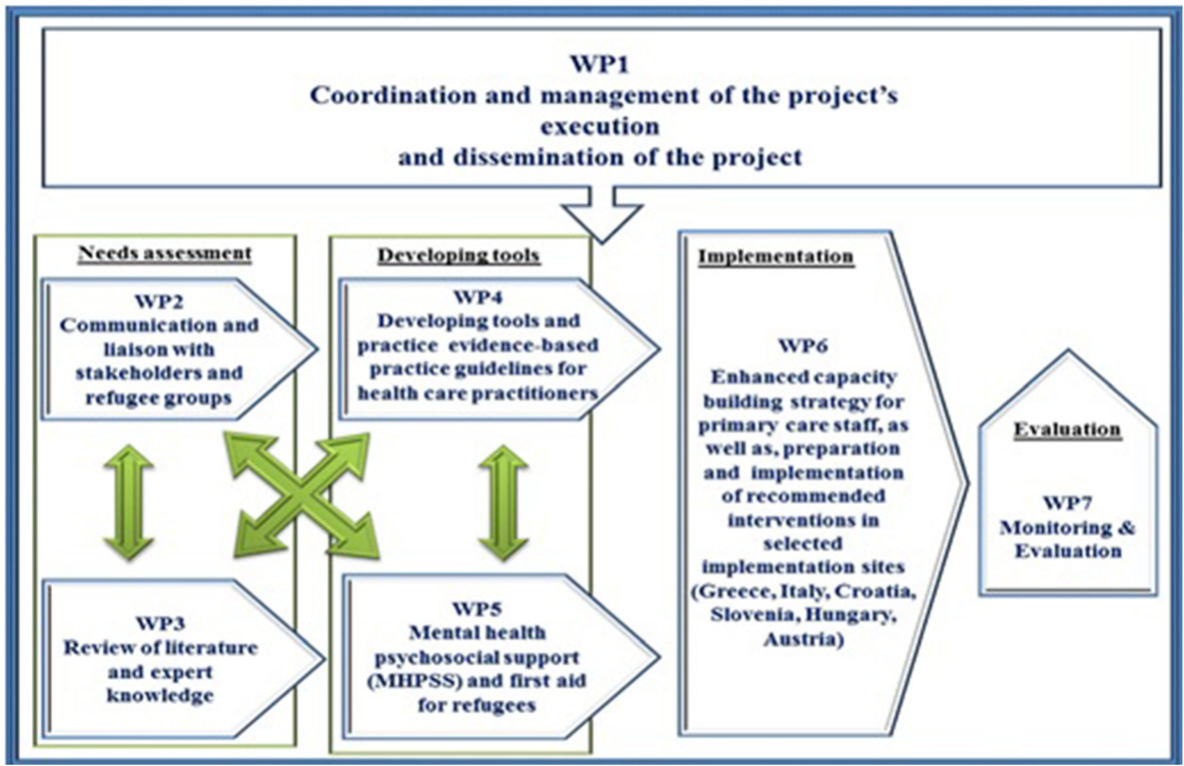
EUR-HUMAN work plan (Source: http://eur-human.uoc.gr/wp-content/uploads/2017/06/Final_Report.pdf)

To meet all abovementioned project objectives, several resources of information had to be incorporated, including a refugee needs assessment, the systematic search of the current literature, and extensive consensus debate about the components of essential tools and interventions for care provision. A graphical representation of the Work Packages of the EUR-HUMAN project is depicted in Fig. 1 , as well as in the Appendix.

### Assessing the refugees healthcare needs by promoting the dialogue with stakeholders, health professionals and refugees

Qualitative research techniques applied in the fieldwork in WP2 were typical for Participatory and Learning Action (PLA) [6]. PLA techniques are inclusive, user-friendly and democratic, generating and combining visual and verbal data. This encourages participation of literate and non-literate stakeholders alike [7]. Visual and written materials were used to explain the topic and to express the opinions, experiences and wishes of the participants. During the sessions, all participants “posted” (using a picture or in writing on a post-it paper) their thoughts and explained them one at a time; the “posts” were categorised with the help of the researchers who acted as facilitators, and recorded on PLA charts in a consistent manner across all sites [6, 8].

This method facilitates the dialogue with national, regional and local stakeholders, as well as with the refugees themselves, in order to assess refugee needs, understand their wishes and elicit their preferences, with the aim of incorporating all these relevant elements in relevant healthcare service delivery. The initiated PLA-brokered dialogue with stakeholders has been anchored in a theoretical framework based on the four constructs of the Normalisation Process Theory (NPT) [9] with the main aim to encourage interactive data generation [6, 10]. “*NPT is a theoretical framework concerned with the work that individuals and organisations have to carry out in order to embed and normalise new, complex ways of working into routine practice*” [11]. It has been used to guide the implementation of system improvements in primary care practice, and alerts researchers and implementers to the realities of implementation in real time and the interactions that do, or do not, occur between the individuals and groups charged with that implementation, by focusing attention on four principal constructs.

Prior to introducing this PLA-dialogue, meetings with stakeholders and healthcare providers were held in order to capture the voice of the refugees and to facilitate the implementation of the project by making all stakeholders aware of the aims and proposed actions and communicating in a transparent manner how their cooperation was critical for timely implementation. A meeting with local stakeholders (representatives from: the municipality and the regional authorities, the hospital, the medical association, PHC services, etc.) took place in the island of Lesvos to that effect. To further facilitate the smooth operationalization of implementing PLA techniques, a two-day training of the PLA actors was arranged. These PLA sessions were held in refugee reception centres between February 2016 and March 2016. During the sessions, cultural mediators or interpreters were engaged to ensure cultural background barriers were eliminated and linguistic requirements, across a wide spectrum of languages, were met. In total, forty-three (43) such training sessions were conducted with ninety-eight (98) refugees and twenty-five (25) healthcare workers, across several countries.

By using the PLA method, a qualitative, comparative case study was performed in hotspots, transit, intermediate -and longer- stay reception centres in seven EU countries (Greece, Austria, Croatia, Hungary, Italy, Slovenia, and the Netherlands) in order to enhance the data collection process. This study was a preliminary effort to assess the current conditions and feasibility of deploying an intervention in the participating countries, while at the same time determining the particular characteristics of each site, which, of course, needed to be taken into consideration. Data have been illustrated on PLA charts. In this manner, it was ensured that verbal and visual forms of data were recorded in a consistent manner across all stakeholder groups. All PLA charts have been digitalised after each data generation session in order to preserve the data. Verbal data have been recorded on post-it notes, in point form or short phrases rather than in full verbatim quotes. All sites analysed their data thematically, individually, on the basis of a universal, for all sites, coding framework provided. All sites reported on their fieldwork using a universal, for all sites, fieldwork evaluation form provided for this purpose. The results of the fieldwork helped to develop the flowchart and the training in Lesvos to address training needs for the local health professionals.

Specific attention was given to the assessment of mental health (MH) needs of refugees, this being the task of a separate dedicated work package of the EUR-HUMAN project. For this purpose, a protocol for the rapid assessment of MH and psychosocial needs of refugees, including tools, guidelines and procedures and interventions for provision of Psychological First Aid (PFA) was developed. This protocol was developed on the basis of a hierarchical approach and according to expert guidelines on addressing the overall approach on MH and Psychosocial Support (MHPSS) (http://eur-human.uoc.gr/work-package-5/). This included practical handbooks, manuals and reports, and validated tools that were identified through a systematic search in the literature. The proposed procedure consisted of triage, i.e., identification of MH conditions requiring immediate specialist attention in the circumstances of very high demand and constrained PHP resources, screening, i.e., identification of individuals who are under increased risk for developing serious MH conditions, immediate assistance based on the PFA principles, and ultimately, referral for full MH assessment and care as needed. This protocol also guided the development of the modules on MH within the online training course and the specific MHPSS face-to-face training. Furthermore, the consortium worked on the development of a protocol for the rapid assessment (RA) of mental health and psychosocial needs of refugees on the basis of an appropriate support model (MHPSS purposes) that was identified through the data collection and review activities (the Model of Continuity of Psychosocial Refugee Care, MCPRC) [12].

### Learning from previous efforts by assessing the evidence found in literature

In addition to this fieldwork, different literature databases were systematically accessed, searched and used for data retrieval during the project to identify relevant literature on suitable interventions, tools and known implementation factors to optimise healthcare provision for refugees in different European settings, focusing on a broad diversity of implementation factors. Search-strings comprising combinations of refugee-related terms and implementation terminology were formulated in English. The search strings were run through six (6) databases (*PsychINFO; Sociological Abstracts; Cochrane; Pilots; PubMed; Embase*). In total, 5492 articles were identified. After discussion, consensus was reached on selecting 264 articles for full text screening. All articles were primarily qualitative, descriptive or reporting on research employing mixed methods.

Furthermore, to strengthen the approach based on evidence, a survey was conducted among almost one hundred (100) participating professionals and experts, which were involved in managing the refugee crisis at the different work locations of the partners in the EUR-HUMAN consortium across countries, i.e., in Austria, Croatia, Greece, Hungary, Italy, and the Netherlands. To the same, effect, ten expert interviews (i.e., UNHCR, the Red Cross, Médecins Sans Frontière (MSF) and Médecins du Monde (MdM) were conducted, to collect information on the context, meaningful structures, process characteristics and challenges of healthcare optimisation for refugees.

### Reaching consensus regarding the tools, guidance and interventions to provide a PHC-focused and patient-centred approach for refugees

Based on the aforementioned, an Expert Consensus Panel from various European countries assembled all the selected and appraised material. The Expert Consensus Panel (ECP) aimed to engage experts in a two-day decision-making process, with the purpose to reach consensus agreement on best practice guidelines, tools and services for the early arrival and longer-term settlement of refugees in European host countries, not excluding groups of refugees that were stranded in “transit” countries for longer periods of time. In total, thirty (30) experts from fourteen (14) different countries attended the meeting. Initially, participants discussed in small thematic groups and then reconvened with the full group to present their conclusions and suggestions and discuss in an extended plenary session. Experts focused on four (4) overarching topics (Linguistic and cultural differences; Continuity of care across sites and countries; PHC team at refugee reception centres; Health promotion information and addressing information needs) and in 5 specific areas **(**Acute illnesses and Triage; Infectious Diseases and Vaccinations; Non-communicable diseases; Mental Health; Mother, Child and Reproductive Health Care). Apart from the experts that were proposed by the consortium partners, a refugee representative also participated.

**Fig. 2:**
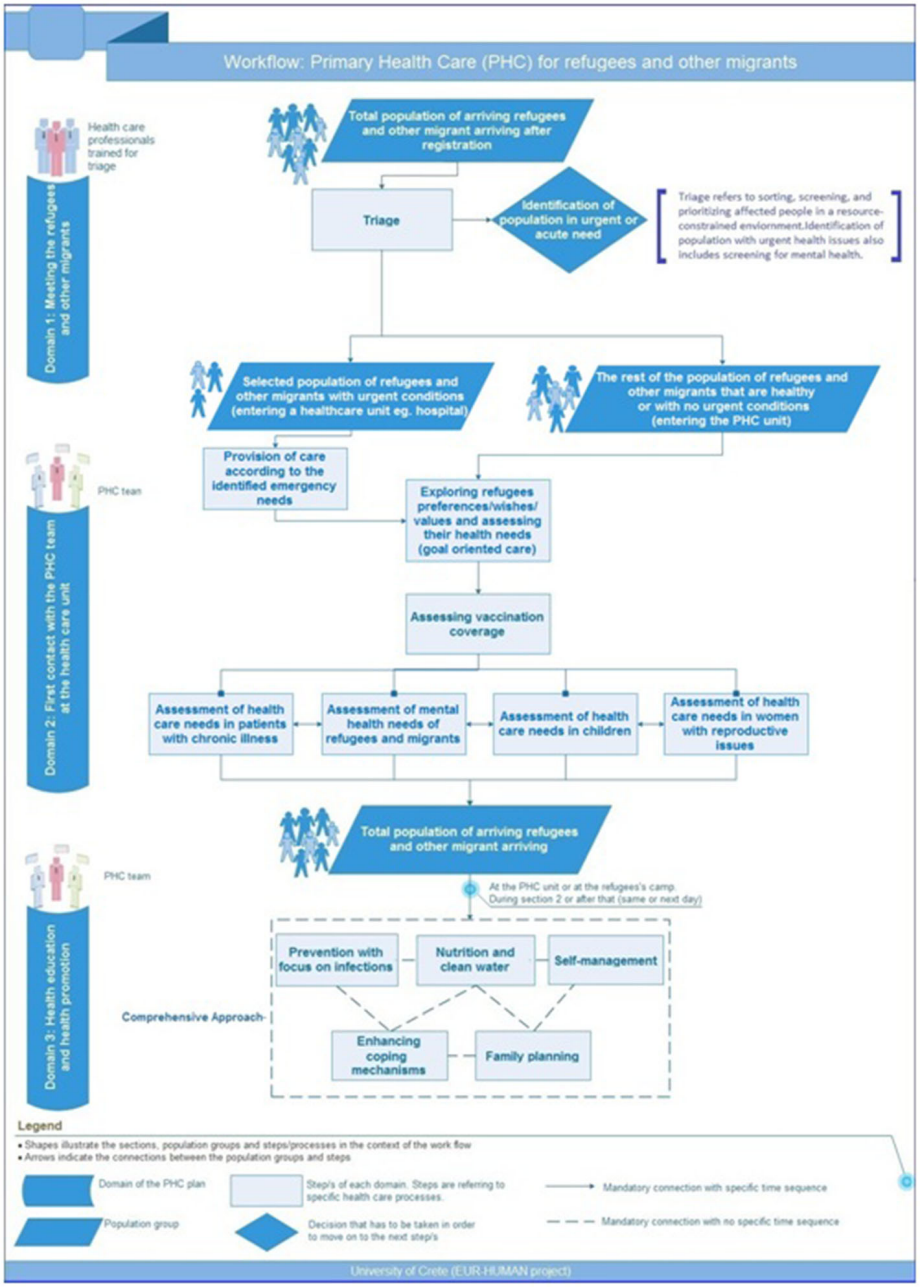
Workflow of Primary Health Care services for refugees and other migrants (Source: http://eurhuman.uoc.gr/wp-content/uploads/2017/06/Final_Report.pdf)

The consensus approved an operational workflow (Fig. 2) to facilitate understanding of the process to be followed.

### Developing evidence-based training material and implementing educational interventions across selected European settings

Based on the information gathered from the different sources including data and information from the PLA approach with refugees, the literature review and interviews with experts, the MHPSS, and insights gained from the output generated through the Expert Consensus Panel guided, the evidence-based training material was made available online. This training material covered eight different areas (modules), namely triage, mental health, communicable diseases, non-communicable diseases, vaccination, mother and child care, cultural and legal issues, and health promotion. Each module consisted of *ex ante* and *ex post* questions in order to evaluate the knowledge gained. It was important to ensure that context-specific parameters were taken into consideration given the high degree of heterogeneity across the settings. Therefore, each country translated and adapted the training material according to their local situation and their needs.

The educational intervention in the six PHC settings was performed through the deployment via an online course in an interactive platform. The core aim of this course is to support -building of the PHC providers by minimising knowledge gaps regarding different issues of PHC for refugees in the respective settings. In addition to this online course in an interactive platform, a face-to-face training carried-out across the different settings in partner countries (Austria, Croatia, Greece, Hungary, Italy and Slovenia). Austria, Hungary and Italy implemented the training material only via the interactive platform, while Croatian, Greek, and Slovenian PHC personnel were trained utilising both approaches, i.e., face-to-face training and interactive online platform. GPs, community nurses, midwives, health visitors/social workers, as well as refugees that were health providers in their country of origin, participated in is this training. Each setting was invited to determine the location and manner, multidisciplinary target group teams and the training topics given the adaptable modular structure of the course in the interactive online platform, as well as to adjust the educational intervention to the healthcare context system context, identified needs and expectations of refugees, and the local the PHC capacity.

This educational intervention was conducted for approximately 1 month. The online course (http://eur-human.uoc.gr/online-courses/) became gradually available from the end of October 2016 onwards and across six (6) implementation settings. People that actually provided health care services to refugees and serve the national healthcare systems were eligible to participate in the course. Course participants were mainly PHC practitioners serving the national health systems or NGOs who deal with refugees. The course is still online, readily accessible via cross-linking in the project website (http://eur-human.uoc.gr/online-courses/). Nearly four hundred (400) primary healthcare workers registered in the course, with more than one third of them having successfully completed the course prior to January 3, 2017 (initial target of 100 exceeded by the first round of training in the context of the EUR-HUMAN project cycle). Most users needed between eight (8) and sixteen (16) hours to complete the full training.

Apart from the educational interventions that they implemented in the six abovementioned European settings, a pilot intervention study was carried out at the Kara Tepe hosting centre of refugees and migrants at the island of Lesvos in Greece. This qualitative approach attempted to identify potential barriers to implementation in real primary care settings, combining what was learned in the educational interventions, including the developed tools, questionnaires and proposed procedures, and to further explore whether the PHC practitioners were better prepared after training. The intervention is targeted to a multidisciplinary team of General Practitioner (GP), nurse, midwife and cultural mediator, a team that was formed to provide healthcare services to them according to their needs. In total, thirty (30) refugees (three (3) men, fifteen (15) women and twelve (12) children) participated in this smaller pilot. All patients received feedback on their health status and recommendations and advice regarding the necessity of the proposed treatment (s) (if any), with further referral to secondary care or specialist care, as needed. The pilot study was evaluated by qualitative research methods (semi-structured interviews, focus groups).

### Evaluating the implemented interventions

The six intervention countries evaluated the selected educational interventions in order to provide answers to questions on their feasibility and acceptability. For this purpose, the NPT method and the NoMaD questionnaire have been utilised [7, 13]. The evaluation procedure took place immediately after the end of intervention with an invitation to the trained PHC personnel to respond to the NoMaD questionnaire. The NoMAD questionnaire is quantitative measure that investigates the implementation process using NPT to evaluate the suggested tools [7, 13]. The NoMAD questionnaire was used to gather respondent views on the implementation of primary care services for refugees in their respective settings. The users of the interactive platform completing the online course were asked to complete an online evaluation survey form, to help assess their experiences regarding the course, determine whether it was useful and of value to respondents, as well as to gather their views on the implementation of primary healthcare services for refugees and migrants in their countries.

Furthermore, recommendations to policymakers were formed on the basis of findings of the EUR-HUMAN project. A meeting with all Consortium partners of the EUR-HUMAN project was held in Crete to conclude the final evaluation and to discuss future actions on establishing a Network for the Care of Refugees in a compassionate manner, and with emphasis on capacity-building actions and MHPSS care.

#### Bioethics

Approval from bioethical committees from all implementing settings (Austria, Croatia, Greece, and Slovenia) has been sought and received according to the existing legislative framework in each participating country (approval was not necessary in Italy and Hungary). In Lesvos, where selected refugees have participated in the pilot intervention study, written information and the respective informed consent form had been provided. Every participant filled in the informed consent form. The informed consent forms were translated into English, as well as Arabic and Farsi, the languages of the countries the majority of refugees were coming from at the time of the project implementation. In the cases where refugees were from a different country and not speaking English or the other two main languages, a translator/cultural mediator informed them. The sessions were audiotaped and transcribed (after requesting and being granted permission). PHC professionals agreed to participate in the training procedure, as well as in its evaluation. Study participation was voluntary for refugees and healthcare professionals alike.

## Discussion

This article describes the implementation of an innovative design to develop tools and educational interventions with the aim of strengthening PHC, indeed it moved to the direction of ‘co-creation’ working with the refugees and other stakeholders. It reports on methodology on how it assembled knowledge from several resources by using evidence-based approaches and through eliciting information from the refugees themselves regarding their needs, wishes, and preferences, but, also, by brokering dialogue with all relevant stakeholders, systematically searching the body of literature and building consensus through bringing together experts from across the EU and in an interdisciplinary fashion. It also focuses on how all these efforts have been translated into training modules and what methodology was used in evaluating its feasibility and acceptability.

Despite several contextual barriers and diverse challenges the EUR-HUMAN project implementation encountered in each of the European settings, it implemented the development and deployment of a context-relevant educational intervention, with a focus on an easy-to-use course in an online interactive platform. This modular course, which was also easy to adapt to local context, was utilised as an instrument of engaging and enabling healthcare providers involved in refugee care, to make knowledge, tools and guidelines available for PHC providers, but, also, other stakeholders. The online platform will continue running beyond the EUR-HUMAN project cycle completion in an effort to contribute overcoming barriers in the provision of high quality, person-centred and integrated healthcare for refugees.

There is currently much discussion on both national and European levels about how the produced material, the output generated by this project, could be utilised on the benefit of care services for refugees and migrants. In Greece, the online course has been given to the authorities of the Second Health Region (the most affected by this crisis in Greece) to disseminate among healthcare personnel, while the UoC team is currently training PHC professionals in the island of Crete; these PHC professionals will be providing services to this vulnerable population. One of the parts of which effectiveness can be ameliorated was the registration procedure, as it was mentioned this was difficult and unnecessarily formal. Another aspect that was considered important for future work, was that of a comprehensive economic evaluation with relevance, on a system level and societal perspective, and extending well beyond economic analysis to policymaking; such an evaluation ought to include the cost of inactivity, degradation of health, longer-term outcomes and many other factors extending well beyond the health services in scope and with an appropriate time horizon for these groups and across geographies.

It is true that this European project met several challenges during its implementation, since many changes in the national policies of the countries where an implementation had been designed and given the impact of the agreement between EU and Turkey in March 2016 on the flow and movement of refugees. It was a capacity-building project that was running in a short period (1 year to satisfy the objectives of all work packages). It is also clear that although the focus of this project was on refugees, other migrants will benefit, as the approach, tools and training is relevant for all these groups and is not limited to refugees. However, a pilot intervention to test how the developed tools and material work in the real world was deployed. Although, this pilot involved a small number of refugees, its findings could be used when future actions and interventions will be debated. Certainly, more specific refugee- or migrant-relevant projects need to be designed. In addition, although the initial EUR-HUMAN project plan to utilise a very robust evidence-based and validated approach, the methods used in this capacity-building project are not considered as the most rigorous ones to create evidence (at least not type A or even B).

The EUR-HUMAN project is anticipated to influence the working conditions and satisfaction of healthcare workers, as well as the interaction and collaboration of the three target groups (refugees; healthcare workers; host communities). These can also contribute significantly to the development and enhancement of capacity building for PHC providers and to promote compassionate first contact, and, ultimately, comprehensive and integrated health care for refugees and migrants.

## Appendix

***EUR-HUMAN Work Packages***

***WP1*** is focused on the overall management and coordination of the project.

***WP2*** utilises relevant methodology including the Participatory and Learning Action (PLA) method to introduce a democratic dialogue with national, regional and local stakeholders, as well as the refugees themselves, to assess their needs, wishes and preferences.

***WP3*** systematically collects and studies the existing literature to identify the factors that promote or hinder the implementation of healthcare interventions for refugees and others migrants in Europe.

***WP5*** aims to develop a protocol for rapid assessment of mental health and psychosocial needs of refugees.

***WP4*** utilises WP2, WP3 and WP5 to synthesise the results during a meeting with an expert panel group in order to guide the development of recommendations and evidence-based practice guidelines.

***WP6*** “translates” all knowledge and guidelines into ready to use training programs for capacity building. Trained health personnel will implement an intervention in selected participating sites.

***WP7*** develops the framework for monitoring and evaluation of the project.

## Abbreviations

CARE: Compassionate Assessment Respecting Expectations
CCM: Chronic Care Model
DPES: Dispositional Positive Emotions Scale
ECP: Expert Consensus Panel
EU: European Union
EUR-HUMAN: EUropean Refugees - HUman Movement and Advisory Network
GPs: General Practitioners
IOM: International Organization of Migration
KOM: Kick off Meeting
MCPRC: Model of Continuity of Psychological Care
MH: Mental Health
MHPSS: Mental Health and Psychosocial Support
M-Ss: Member States
NPT: Normalization Process Theory
PFA: Psychological First Aid
PHC: Primary Health Care
PLA: Participatory Learning & Action
POE: Port of Entry
RA: Rapid Assessment
WHO: World Health Organization
WP: Work Package
